# The roles of jim lovell and uninflatable in different endopolyploid larval tissues of *Drosophila melanogaster*

**DOI:** 10.1371/journal.pone.0237662

**Published:** 2020-08-21

**Authors:** Fanli Zhou, Stephanie R. Green, Michael Tsay, Safina Hsu, Rami Dibbs, Kathleen M. Beckingham

**Affiliations:** 1 Biosciences Dept, Rice University, Houston, Texas, United States of America; 2 Data Science Dept, University of British Columbia, Vancouver, British Columbia, Canada; 3 McGovern Medical School, UT Health Science Center at Houston, Houston, Texas, United States of America; 4 UTHealth School of Public Health, Houston, Texas, United States of America; 5 Louisiana State University School of Medicine, New Orleans, Louisiana, United States of America; Centre National de la Recherche Scientifique, FRANCE

## Abstract

The larvae of *Drosophila melanogaster* grow rapidly through use of a highly truncated cell cycle in which mitosis is entirely eliminated. The *Drosophila* homolog of the protooncogene transcription factor Myc plays a major role in promoting this endopolyploid (EP) growth. We have previously determined that the gene *jim lovell* (*lov*), which encodes a member of the BTB/POZ (**B**ric-a-brac, **T**ramtrack, **B**road/**Po**x virus **z**inc finger) domain family of transcription factors, is also required for EP growth in one larval tissue, the trachea. Here we show that *lov* promotes EP growth in three further tissues indicating a fundamental role in this process. However, epistasis experiments revealed heterogeneity in *lov*’s action in these tissues. Whereas in the tracheae and salivary glands *lov* acts downstream of *Myc*, in the fat body, reduced expression of *lov* does not impede the action of *Myc*, indicating an upstream action for the gene. We show here that *lov*’s regulation of the gene *uninflatable (uif)* in the tracheae is a component of this difference. *uif* is required for tracheal EP growth downstream of *Myc* and *lov* but has no equivalent role in the fat body. Although Uif is a transmembrane component of the plasma membrane in the tracheae, its action downstream of Myc suggests an intracellular role for the protein in the tracheae. In addition to regulating *uif* expression in some tissues we also show that *lov* locates to the nucleolus, indicating it can function in both polymerase I and polymerase II transcriptional events. Our major finding is that tissue-specific mechanisms can interact with universal growth promotion by *Myc* to generate the individual endopolyploid organs of the larvae.

## Introduction

Within all multicellular organisms, mitotic cell cycling is the dominant mechanism for producing tissue and organismal growth. However, variant modes of growth exist in which supernumerary copies of the genome, contained in one or more nuclei, are generated within a single enlarged cytoplasm [reviewed in 1, 2]. These variant mechanisms are seen across evolution and have long been known to play roles in tissue differentiation. However, more recently some variants have been recognized as inducible growth phenomena activated by environmental stresses. In particular the ability of some cancer cells to resist apoptosis in response to drug-induced DNA damage involves transition to a polyploid state. Subsequently, a few cells can then revert to mitotic metastatic growth [reviewed in 3, 4]. In the variant growth mechanisms collectively termed endocycling (EC), rounds of genome replication occur without mitotic cytoplasmic division so that giant cells are generated. In the most extreme form of EC, here termed endopolyploid (EP) growth, the entire cell cycle M phase is absent, including nuclear envelope breakdown and chromosome segregation: the cell cycle consists solely of rounds of synchronized DNA synthesis (S) alternating with gap (G) phases [1, 2, 5]. Thus in EP growth, giant cells with giant polyploid nuclei are generated.

Giant cells synthesize fewer cell surface components than an equivalent mitotic cell mass. In addition, skipping elements of M phase saves both cellular resources and time. As a result, EC mechanisms are often associated with the need for rapid growth. EP growth, in which M phase is completely absent, offers the greatest potential for a rapid growth rate amongst the EC mechanisms. The major function of the larval stage in the *Drosophila* life cycle is that of extremely rapid growth. Over a four day period, larvae increase 200 fold in weight [6] and their size at pupation defines that of the final adult. With the exception of a few tissue types (in particular, the imaginal discs and most of the nervous system) all of the larval tissues grow by an EP mechanism [7]. The *Drosophila* larva thus offers an opportunity to examine the coordinated deployment of EP growth in multiple tissues and thus to determine whether differences in the regulation of EP growth exist in differing cell types.

Although the signaling pathways that initiate EP growth in the larva are not well characterized, it is clear that the single Myc protein of *Drosophila* is a major downstream positive regulator of EP growth in the larval tissues [8]. Failed EP growth has been directly demonstrated in three *Myc* null larval tissues—salivary glands, fat body, and hindgut [8]—and the essentially complete absence of growth in *Myc* null larvae argues for a regulatory role in all EP tissues [8]. Endoreplication of the nuclear DNA and overall cellular growth are tightly coordinated in EP growth. However, available evidence suggests that Myc does not directly affect DNA replication but rather acts to stimulate cellular growth by multiple genome-wide actions including increased ribosome synthesis, mRNA translation capacity and overall cellular metabolism [9–13]. Myc is a bHLH class transcription factor and some of these changes are direct effects on transcription as a result of Myc binding to DNA, with or without its binding partner, Max [9,14]. But other effects are via indirect action. Most notably in *Drosophila*, in marked contrast to the situation in mammals [15], Myc stimulation of 18S and 28S rRNA production does not involve enhanced Polymerase I transcription through direct binding of Myc to rRNA gene promoters [10]. Instead, indirect actions of Myc such as enhanced production of Polymerase I co-factors and rRNA processing enzymes [reviewed in 16] stimulate rRNA synthesis. It has been proposed that the Myc-induced changes in ribosome levels and translation rates may increase the protein levels of critical components of the S to G endocycle and thus produce the tight linkage between increased growth and faster DNA endoreplication seen in EP tissues [18].

Ribosome synthesis is orchestrated in the nucleolus where the rDNA arrays are transcribed and ribosome assembly proceeds within the granular components of the organelle. In *Drosophila* larval tissues, *Myc* overexpression greatly increases the size of the nucleolus [8], but in keeping with its indirect role in rRNA production, Myc protein localizes to many euchromatic sites on the larval salivary gland polytene chromosomes but not to the rDNA arrays [17]. Several of the *Drosophila* genes identified as being positively transcriptionally regulated by Myc encode nucleolar proteins and two nucleolar components—a DEAD box helicase (Pitchoune) [18] and a protein of unknown function (Nol12/Viriato) [19]–are indicated to act genetically downstream of *Myc* in EP growth.

The Jim Lovell protein (Lov), previously known as Tkr, is a member of the Tramtrack (Ttk) subset of BTB/POZ proteins in *Drosophila* [20]. In addition to a single BTB/POZ domain, which acts as a protein interaction interface, most of these proteins contain one or more DNA binding motifs—either of the zinc finger or pipsqueak category [21]. Lov contains a single pipsqueak domain. Like most of the Ttk subgroup, Lov is thus implicated in transcriptional regulation.

We initially isolated the *lov* gene through its role in gravitaxic behavior in adult *Drosophila* [22] which led to our naming it for the heroic Apollo 13 astronaut, Jim Lovell. We subsequently identified additional behavioral abnormalities through mutant analysis and *lov* RNAi knockdown. Surprisingly the hypoxic behaviors we discovered in larvae with tracheal *lov* knockdown proved to originate from fluid filling of the tracheae due to strong inhibition of tracheal EP growth. This finding led us to the investigation of *lov* function in other endopolyploid larval tissues presented here. We have found that Lov localizes to the nucleolus and regulates EP growth in all the tissues examined. However, epistatis experiments for *lov* and *Myc* revealed that *lov* regulates EP growth differently in different tissues. In the tracheae, *lov* positively regulates *uninflatable (uif)*, a gene encoding a transmembrane protein of the tracheal cell apical surface [23] which acts downstream of *Myc*. In contrast, *uif* has no role in fat body EP growth and the actions of *lov* in this tissue are all upstream of *Myc*. These findings uncover heterogeneity in the mechanisms used to orchestrate EP growth throughout the organism.

## Material and methods

### Drosophila stocks and crosses

Canton-S was used as the wild type (+) stock for this work. Stocks carrying constructs and mutations used in these studies are given in S1 Table. Additional genotypes were prepared from these stocks by standard genetic crosses using dominant markers and balancer chromosomes. Stocks were maintained on standard cornmeal/yeast/agar food at 220C and 180C. Strains are available upon request. See [24] and [23] for initial characterization of the *lov* RNAi and *uif* RNAi stocks used here.

### Generation of staged larvae

Crosses to generate larvae of appropriate genotypes were performed as described previously [25]—but using grape, not apple, juice plates. To generate staged larvae, four hour egg collections were performed and the resulting larvae were grown in uncrowded conditions in yeast mounds atop grape plates.

### Semi Q RT-PCR

RNA preparation, cDNA synthesis and gel electrophoresis were performed as previously [20]. Emerald Amp GT PCR Master mix (Takara Biotechnology Ltd) was used for PCR reactions (30 cycles) using appropriate cycling conditions in an Eppendorf Mastercycler. Two or three separate RNA preparations were examined for each genotype. Primers used for PCR reactions were *Actin* 57B—forward

5’ TTCCAAGCCGTACACACCGTAACT 3’, reverse 5’ TCATCACCGACGTACGAGTCCTTCT 3’
*uif* set 1 forward 5’ ATCAAGCACTCGTGGGATAAA 3’, reverse 5’GTCCTGGAACTGCAGGATAAT 3’
*uif* set 2 forward 5’ CAACTCCTGAGCGACAAGAA 3’, reverse 5’CTGATAGCGAGTGTCCACAAA 3’

### Tissue staining

Tissues were fixed in 3% paraformaldehyde in PBS for 30 min then immunostained as described previously [20]. Mouse anti-Coracle and anti-Armadillo antibodies (Developmental Studies Hybridoma Bank (DSHB)) and anti-Fibrillarin (Thermo Fisher Scientific) were used at 1:10, 1:400, and 1:400 dilution respectively. Goat anti-mouse IgG labeled with Alexa Fluor 594 (BD Biosciences) was used at 1:500 dilution as the secondary antibody. Guinea pig anti-Lov antibody, prepared and characterized as described previously [20], was used at a 1:50 dilution followed by goat anti-guinea pig IgG labeled with Alexa Fluor 594 or 488. DNA was stained with DAPI (4’,6-diamidino-2-phenylindole) at 1μg/ml prior to mounting in 70% glycerol. Fat body was simultaneously fixed and stained in 37% formaldehyde containing five units/ml of Oregon-Green 488nm Phalloidin (Molecular Probes) and DAPI at 1μg/ml.

### BrDU incorporation

Staged larvae three days after egg lay (AEL) were fed on BrDU (1mg/μl in PBS) for 24 hours after which salivary glands were dissected out, washed, fixed, and immunostained with anti-BrdU antibody (G3G4, DSHB 1:10 dilution) followed by goat anti-mouse Alexa Fluor 594 nm tagged secondary antibody.

### Transmission electron microscopy

Samples were fixed with 2% paraformaldehyde, 2.5% glutaraldehyde, and 0.13M sucrose in 0.1M cacodylate buffer for 24 hours at 40C, followed by post fixation with 1% osmium tetroxide in 0.1M cacodylate for an hour. After dehydration, samples were treated progressively over 24 hours with 1:1 ethanol:Spurr’s resin (1:1 E:S), 1:3 E:S, and finally four changes of 100% Spurr’s resin and then incubated at 620C overnight. Sections were stained with uranyl acetate and lead citrate and imaged on a Hitachi Model H7500 Transmission Electron Microscope.

### Larval body length measurements

Staged larvae were reared for three or six days AEL, depending on the genotype and timing of any larval death. After collection, larvae were immobilized on microscope slides by brief exposure to 700C on a hot plate in 70% glycerol. Coverslips were then added and images taken on an Olympus SZX12 microscope. NIH Image J was used for length measurements. A millimeter scale ruler was used to calibrate the program and larvae were measured from the tips of their snouts to the ends of their spiracles. The “Segmented Line” tool, which allows measurement of curved lines, was used for all measurements. With this protocol, Image J gave reproducible measurements in millimeters to greater than one decimal place accuracy.

### Larval burrowing and tunneling behavior

These behaviors were assayed using our published protocol [26].

### Quantitation of tissue, cell, nuclear and nucleolar size

Tissues were flattened and quantitation was therefore mainly expressed as tissue, cell, nuclear, or nucleolar areas. For some later work, after acquisition of Imaris software, nuclear measurements were calculated as volumes.

#### Salivary glands

*forkhead*-Gal4 (*fkh*-Gal4) and *pumpless*-Gal4 (*ppl*-Gal4) were used for salivary gland expression. These constructs produced comparable results and were used interchangeably. Glands were dissected from staged third instar larvae. After staining with anti-Armadillo antibody (to delineate cell membranes) and DAPI (to stain nuclei), glands were mounted in 50 μl 70% glycerol on microscope slides and imaged with a Zeiss Axioplan 2 microscope. Images at multiple depths were taken to ensure capturing optimal focal planes for all cells and nuclei measured. Components of the tissue were then quantitated using NIH Image J calibrated with a suitable scale. The Polygon Selection tool, which allows imaging of curved areas was used. For salivary gland lobe size, Differential Interference Contrast images were quantitated. Measurements of cell and nuclear size were limited to the distal one third of the glands.

#### Epidermis

The epidermis was dissected from staged larvae carrying the A58-Gal4 UAS-*src* GFP, UAS-nuclear RED chromosome at five days AEL. The protocol described previously was used [27] but larvae were opened from the dorsal side. After fixation, the tissue was placed on a slide in 50 μl 70% glycerol and flattened under a coverslip for imaging using an Axioplan 2 microscope. Images were taken in three regions—between the 3rd/4th, 4th/5th and 5th/6th denticle belts. Tendon cells interdigitated into the ventral epidermis were identified with *stripe*-Gal4 >mRFP. Their nuclei are noticeably smaller than those of the epidermis proper allowing them to be excluded from analysis. Cell and nuclear areas were quantitated using NIH Image J.

#### Fat body

*Apolipophorin*-Gal4 (*Lpp*-Gal4) was used to give fat body expression. Staged larvae were grown on yeast paste, using mounds of 0.8 g to support groups of 10 larvae. The fat body tissue surrounding the larval gonad was dissected from larvae at five days AEL. After fixation and DAPI staining, tissue was mounted in 50 μl 70% glycerol for imaging on a Zeiss AxioImager M2 microscope. Nuclear areas were measured using an algorithm generated within the Surface Area Module of Bitplane Imaris software version 9.2.1. Data were then exported to Microsoft Excel and the mean and SEM of each data set were used to generate distribution graphs for each genotype.

#### Tracheae

*btl*-Gal4 and *cut(ue)*-Gal4 were used to express constructs in the tracheae. Tracheae were dissected from staged larvae at six days AEL, stained with anti-Armadillo antibody and DAPI and mounted in glycerol as described previously [24]. Confocal images were acquired on a Zeiss 710 LSM confocal microscope and nuclear volumes were calculated using the volume module of the Bitplane Imaris software.

## Results

### *lov* plays a role in the EP growth of multiple larval tissues

Lov is a nuclear protein with complex expression patterns during embryogenesis, indicating multiple roles in development [20]. No immunostaining for Lov is detected in the embryonic tracheal system up to the late stages of embryogenesis [20] but Lov stains the larval tracheal nuclei strongly [24], suggesting a role in larval tracheal function. We used the Gal4-UAS system [28] to address this possibility. The *cut* (*ue*)-Gal4 driver, which expresses just in metamere 10 of the dorsal trunk tracheae [24] was used to drive a *lov* RNAi construct shown to specifically reduce *lov* transcript levels [24]. We found that *lov* knockdown with this RNAi line severely inhibited EP growth in this tracheal metamere, resulting in extreme shortening of the segment, fluid accumulation throughout the lumen, and often, tracheal breakage at the junction of metamere 10 with the spiracles. Tracheal fluid accumulation produced hypoxic behavior in the larvae, giving us a behavioral diagnostic for the inhibition of EP growth [24]. Expression of *lov* RNAi throughout the tracheae with *breathless* (*btl*)-Gal4 caused fluid filling of the tracheae after hatching followed by early death. We have recently determined that *lov* null mutant larvae show similar tracheal damage (S1 Fig), confirming the specificity of the RNAi construct.

**Fig 1:**
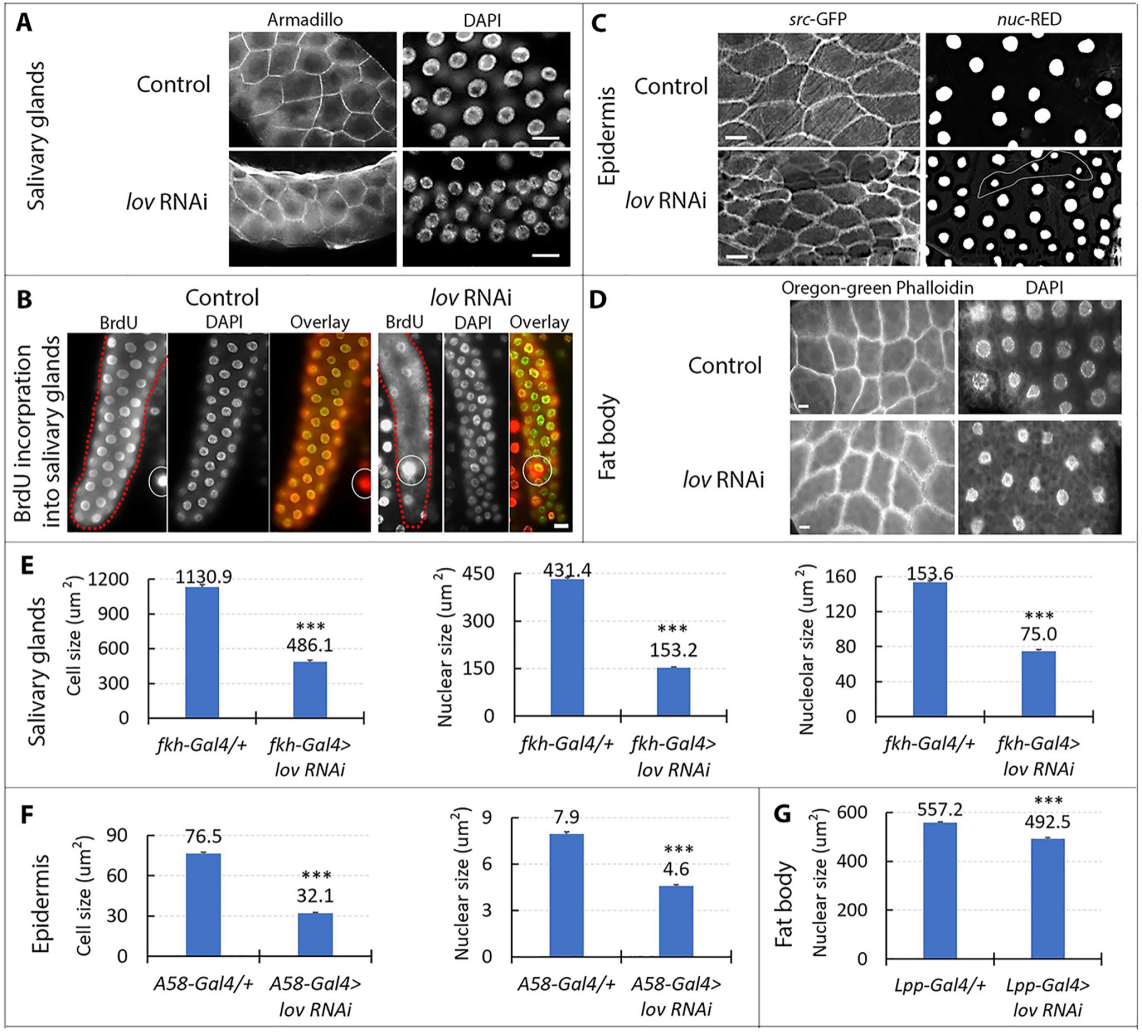
*lov* plays a role in EP growth in multiple larval tissues. A. *lov* RNAi knockdown in larval salivary glands. Control = *fkh*-Gal4/+, *lov* RNAi = *fkh*-Gal4 > *lov* RNAi. Parts of three nuclei of the fat body attached to the salivary gland are shown in the upper part of the *lov* RNAi DAPI panel. The Armadillo antibody does not stain the fat body and therefore the rest of the fat body tissue is not visible. B. *lov* knockdown decreases endoreplication in the salivary gland. Overlays show BrdU immunostaining in red and DAPI staining in green. The large nuclei (examples are circled) with strong BrdU immunostaining are in the fat body tissue attached to the salivary glands. Control = *ppl*-Gal4/+, *lov* RNAi = *ppl*-Gal4 > *lov* RNAi. C. *lov* RNAi knockdown in the larval epidermis reduces EP growth. The A58-Gal4 driver chromosome carries UAS-*src*-GFP and UAS-nuc-RED to stain the plasma membranes and nuclei, respectively. Tendon cells, with smaller cells and nuclei, were found amongst the epidermal cells (see four outlined nuclei in *lov* RNAi right panel). D. *lov* RNAi knockdown in the fat body reduces nuclear size. Control = *Lpp*-Gal4/+, *lov* RNAi = *Lpp*-Gal4 > *lov* RNAi. Scale bars in A.- D. = 20 μm. E. Effects of *lov* knockdown on salivary gland cell, nuclear, and nucleolar size. At least 10 larvae were examined for each measurement. At least 100 cells, 250 nuclei and 375 nucleoli were quantitated for each genotype. *** p< 1x10^-8^ for all three data sets in Student’s t test. F. Effects of *lov* knock down on epidermal cell and nuclear size. 150 cells from 10 individuals were measured for each genotype. For each individual, measurements were made in three regions of the epidermis. To exclude tendon cells only the five largest cells/nuclei were measured in each region. *** p for cell size = 2.26x10^-111^, ****p for nuclear size = 1.14x10^-53^. G. Effects of *lov* knock down on fat body nuclear size. Tissue from 10 individuals and 450 nuclei were measured. *** p = 1.47x10^-22^.

To determine whether *lov* might have a general role in larval EP growth, we used appropriate Gal4 lines to drive *lov* knockdown in the salivary glands, epidermis and fat body (see Material and methods). Given the multiple metabolic roles of the fat body [29], which could involve regional functional differences in the tissue, we used a specific segment of the fat body—that associated with the larval gonad—for our experiments. *lov* RNAi expression produced significantly smaller nuclei in all three tissues (Fig 1A–1G) and in the salivary gland and epidermis cell size was also consistently reduced (Fig 1A, 1C, 1E and 1F). However, we could not identify an effect of *lov* RNAi on cell size in the fat body (Fig 1D). In the salivary glands, we examined BrdU incorporation into nuclear DNA in the late stages of EP cycling to determine whether *lov* knockdown also affected DNA endoreplication. As shown in Fig 1B, *lov* knockdown greatly decreased BrdU incorporation indicating that the decreased nuclear size reflects reduced endoreplication.

### Lov localizes to the nucleolus in larval salivary gland polytene nuclei

**Fig 2:**
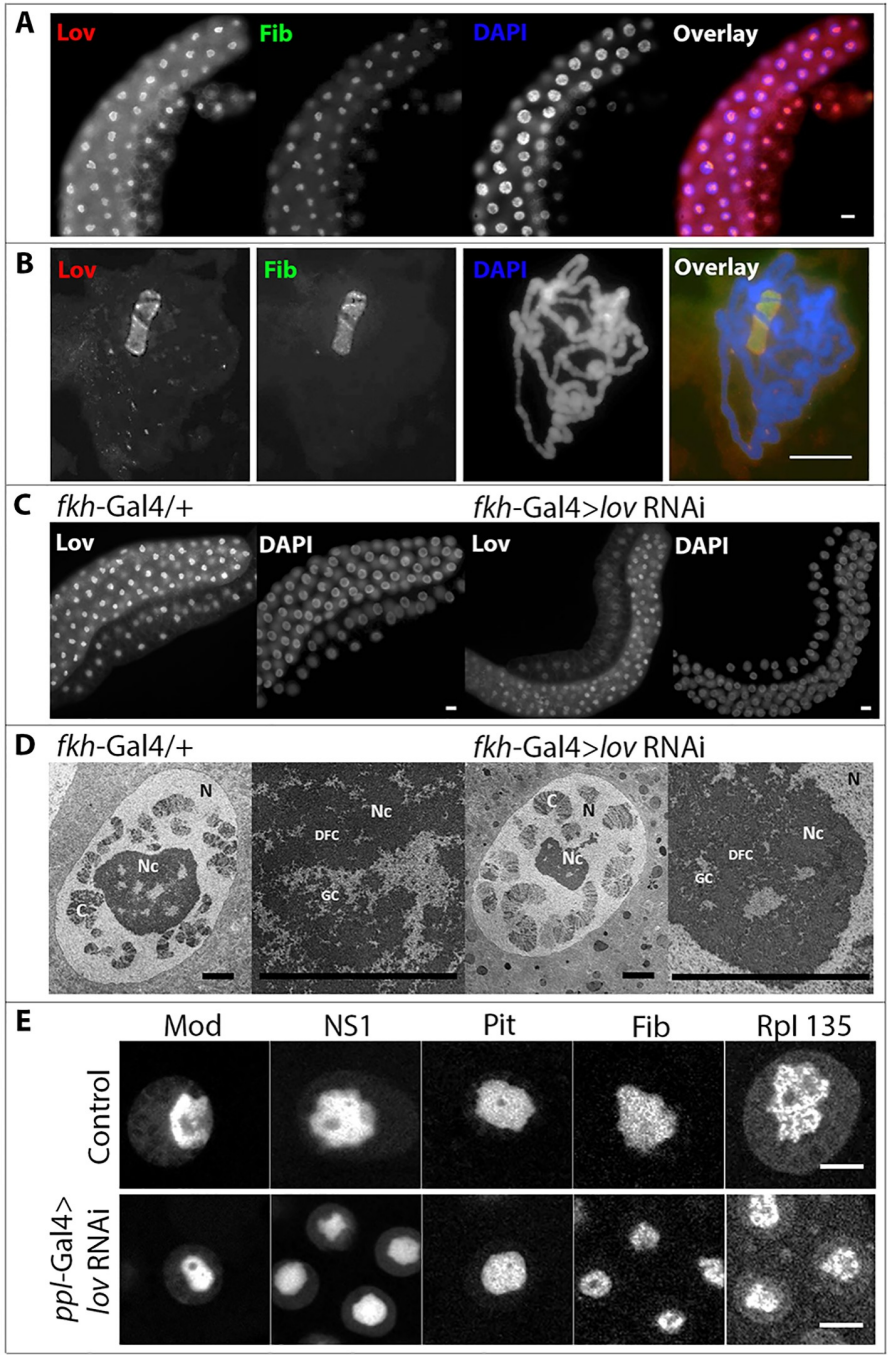
Lov is a nucleolar protein. A. Lov co-localizes with Fibrillarin, a known nucleolar protein, in salivary gland nuclei. Whole mount staining of a salivary gland. Scale bar = 20 μm. B. Lov co-localization with Fibrillarin in a salivary gland polytene chromosome squash. Scale bar = 20 μm. C. lov knockdown in the salivary glands reduces Lov protein in the nucleolus. Lov immunolocalization appears as smaller, weaker, patches of nuclear stain in fkh-Gal4 > lov RNAi salivary glands than in control fkh-Gal4/+ glands. Scale bar = 20 μm. D. lov knockdown produces smaller nucleoli without altering nucleolar composition. TEM images of control and lov RNAi salivary gland nuclei. Both nuclei (N) and nucleoli (Nc) are smaller in lov RNAi cells (panels 1 and 3) but nucleoli remain composed of fibrillar (DFC) and granular (GC) components (panels 2 and 4). C = chromosomes. Scale bars = 5 μm. E. lov knockdown does not alter localization of multiple nucleolar proteins. Localization of EGFP-Modulo (Mod), EGFP-Nucleostemin 1 (NS1), Pitchoune-EGFP (Pit), RFP-Fibrillarin (Fib) and RNA polymerase 1 subunit 135-EGFP (Rpl 135) in nuclei/nucleoli from control and lov RNAi salivary glands. Although lov knockdown decreases the size of the nuclei and nucleoli, the levels and distribution of these proteins in the nucleoli appear similar to those in control tissue. Scale bars = 10 μm.

As a route to identifying potential targets for *lov* we performed Lov immunolocalization to the larval salivary gland cell polytene chromosomes. Surprisingly, the major Lov binding site proved to be the giant nucleolus in these cells (Fig 2A and 2B), as confirmed by co-staining with Fibrillarin, a known nucleolar protein [30]. Accumulation of Lov in the nucleoli could be detected even in whole cells of EP tissues: the Lov immunostaining localized to a sub-compartment of the nucleus that stains poorly for DNA but strongly for fibrillarin (Fig 2A). Lov nucleolar staining was clearly reduced in *lov* knockdown tissue (Fig 2C).

The *Drosophila* nucleolus is atypical in that a fibrillar center (FC) seen in higher organisms and believed to contain inactive chromatin and stored proteins, is not detectable [31, 32]. The nucleolus is thus largely composed of the dense fibrillar (DFC) and dense granular (GC) components present in other species and associated with rRNA transcription/processing (DFC) and ribosome assembly (GC) [32]. These elements are loosely organized and intermingled in the nucleolus in a manner that varies from cell to cell. Both the nuclei and nucleoli in salivary glands are smaller when *lov* expression is inhibited in the salivary glands (Fig 1E for quantitation and Fig 2C and 2D) and in the other EP tissues examined. However TEM analysis of salivary gland nucleoli revealed that the overall organization of the nucleoli into DFC and GC components is not affected by loss of *lov* function (Fig 2D).

To gain further insight into *lov*’s role in the nucleolus, we examined the localization of five fluorophore-tagged nucleolar proteins upon *lov* RNAi knockdown in salivary glands (Fig 2E). These were i) Fibrillarin, a 2’ methyl transferase for rRNA and a component of several small nucleolar RNPs required for rRNA processing [33]; ii) Nucleostemin 1, a GTPase required for release of the large rRNA molecules [34]; iii) RNA polymerase I subunit 135; iv) Pitchoune, a DEAD box family ATP dependent RNA helicase [18] and v) Modulo, the *Drosophila* Nucleolin homolog, which has multiple roles in rRNA production [35]. For all five proteins, neither their presence in the smaller nucleoli of the lov knockdown tissue, nor their distribution within them, was detectably altered by loss of *lov* function (Fig 2E).

These findings suggest that Lov is not a structural protein with a fundamental role in organizing the nucleolus. But they do show that Lov is required for production of a normally sized nucleolus. Of the two other *Drosophila* nucleolar components known to be regulated by Myc in EP growth, Pitchoune, which acts in rRNA processing, also behaves as a non-structural protein [18]. In contrast, Viriato clearly has a structural role: alteration of Viriato levels has profound effects on organization of the nucleolar compartments [19].

### Epistatic studies reveal tissue heterogeneity in the interactions of *lov* and *Myc*

The nucleolar association of Lov and its widespread involvement in larval EP growth are highly reminiscent of the actions of Myc. We therefore set out to determine the relative order of action of *lov* and *Myc* in this process. Our approach was to use the opposing phenotypes of *lov* RNAi knockdown (decreased EP growth) and *Myc* over-expression (enhanced EP growth) [16] and to determine which phenotype proved epistatic. The complementary analysis,—examination of the combined phenotype of *lov* over-expression and *Myc* knockdown—could not be performed because, in contrast to *Myc*, *lov* over-expression produces a more severe version of the *lov* under-expression phenotype: EP growth in all tissues examined is more strongly suppressed producing even smaller cells with smaller nuclei and nucleoli, and inducing earlier, larval death when over-expressed in the tracheae and epidermis (see Discussion for further consideration of this phenotype).

We performed epistatic analysis in three EP tissues, the trachea, the salivary gland and the fat body, using tissue specific Gal4 drivers to simultaneously drive both the *lov* RNAi and UAS-*Myc* constructs. These experiments involved comparing the effects of a particular Gal4 construct driving a particular UAS construct either alone (here UAS-*lov* RNAi or UAS-*Myc*) or in the presence of a second UAS construct (here, co-expressed UAS-*lov* RNAi and UAS-*Myc*). In such experiments, the phenotype of either UAS construct could be diminished as a result of competition for a limited supply of Gal4. Currently, two major classes of UAS construct are used in *Drosophila*: those with five, and those with 10, copies of UAS, and our experiments required use of combinations of both types of construct. We therefore included controls for all the Gal4 drivers used here to determine whether enough Gal4 was produced to avoid competition effects. In these control genotypes, a neutral second UAS construct (GFP, mRFP or mCherry) with the appropriate UAS copy number was present.

For the tracheae, examining the *Myc* /*lov* RNAi interaction at the cellular level proved challenging. In our previous studies [24] we used both *btl*-Gal4, which expresses throughout the tracheal system, and *cut*(*ue*)-Gal4, which expresses specifically in metamere 10, to knock down *lov* in the tracheae. *btl*-Gal4 > *lov* RNAi larvae die at a stage when they are too small for dissection. Expressing *lov* RNAi specifically in tracheal metamere 10 with *cut(ue)*-Gal4 allows larvae to survive better but *lov* knockdown in this small tracheal region produces twisting and mechanical tracheal breakage as a result of the severe growth inhibition, making imaging difficult. Although we were able to image metamere 10 cells consistently in *cut*(*ue*)-Gal4 > *lov* RNAi larvae in our previous work [24] greater tracheal distortion in *cut*(*ue*)-Gal4 > UAS-*Myc*; *lov* RNAi animals prevented reliable imaging. We therefore assessed epistasis between *lov* and *Myc* in the tracheae through behavioral and growth assays.

**Fig 3:**
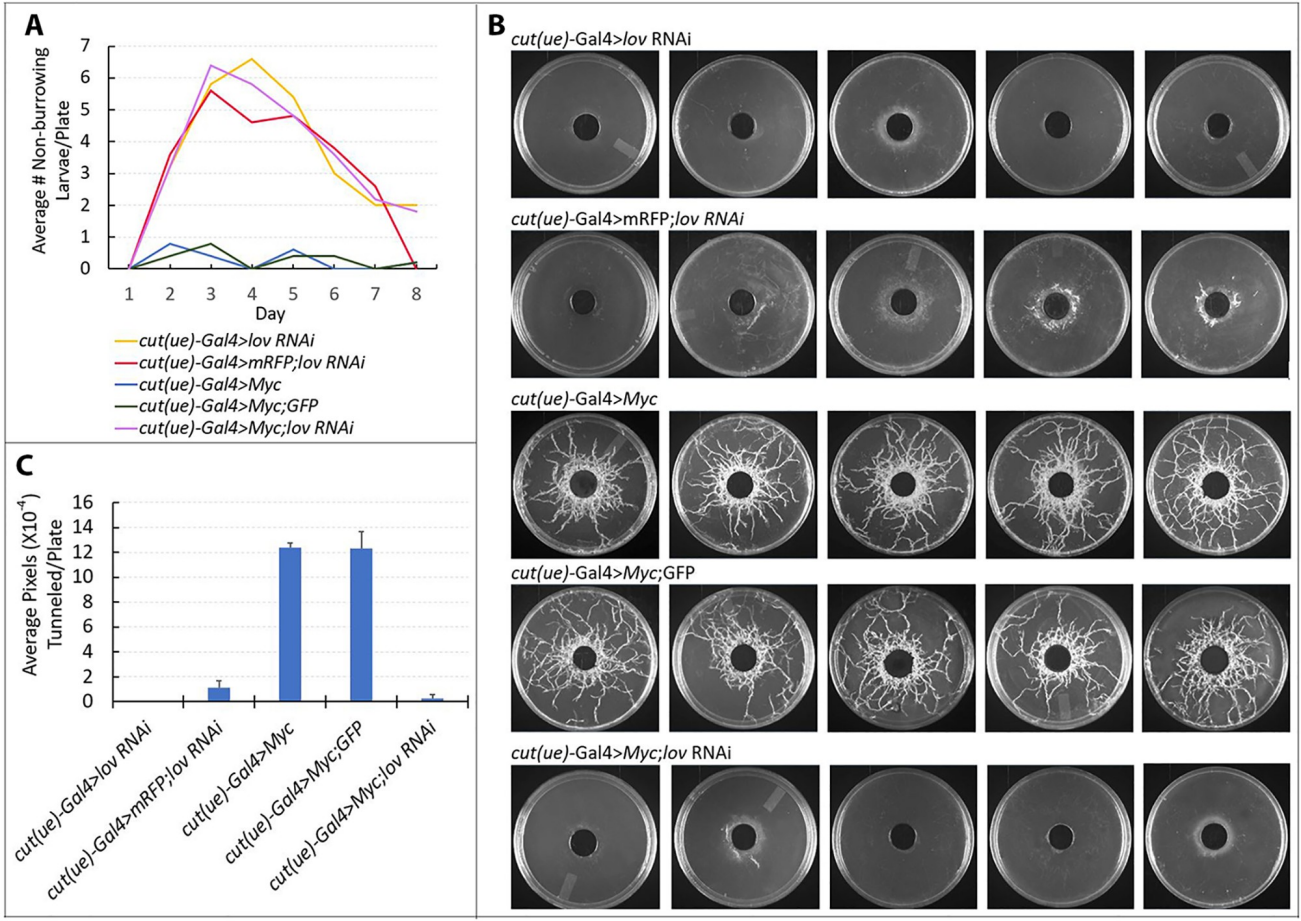
*lov* acts downstream of *Myc* in larval behavioral assays. *cut(ue)*-Gal4 > *lov* RNAi larvae are hypoxic and fail to burrow into their food and to tunnel in a substratum during the wandering phase. In contrast, *cut(ue)*-Gal4 > *Myc* larvae show normal burrowing and tunneling. Larvae (10 per plate) were assayed to determine whether the *lov* RNAi or *Myc* phenotype dominates. Petri plates with a soft agar layer and a central hole filled with yeast paste food were used as in our published protocol [26]. A. Burrowing activity. *cut(ue*)-Gal4 > *lov* RNAi larvae (~60% total) are outside their food during larval feeding. Numbers decline as larvae transition to pupae. The behavior of the test *cut(ue)*-Gal4 >*Myc*; *lov* RNAi larvae is very similar to that of *cut(ue*)-Gal4 > *lov* RNAi larvae. The behaviors of two control genotypes (*cut(ue*)-Gal4 >mRFP; *lov* RNAi and *cut(ue)*-Gal4 > *Myc*; GFP (each 15 copies UAS total) establish that Gal4 levels are not limiting in the test larvae. B. Tunneling behavior. Assay plates were imaged to reveal tunnels produced during wandering phase. *cut(ue)*-Gal4 > *lov* RNAi larvae show a complete absence of tunneling and larvae with the added presence of 5xUAS-mRFP have very similar activity. The test *cut(ue)*-Gal4 > 5xUAS-*Myc*; *lov* RNAi larvae show the minimal tunneling seen for the *cut(ue)*-Gal4 > 5xUAS-mRFP; *lov* RNAi control larvae. C. Quantitation of tunneling behavior. NIH Image J was used to quantitate tunneling in the assay plates shown in B.

The tracheal damage in *cut(ue)*-Gal4 > *lov* RNAi larvae results in fluid entry and organismal hypoxia which produces highly diagnostic hypoxic behaviors: larvae fail to burrow into their food while growing and fail to tunnel into a substratum while wandering [26]. In contrast, although *cut(ue*)-Gal4 > *Myc* larvae show enhanced EP growth in tracheal metamere 10, the tracheae remain air-filled and they show normal burrowing and tunneling behavior. Thus we could ask which set of behaviors dominates in *cut(ue)*-Gal4 > *Myc*; *lov* RNAi larvae. The control genotypes to check for Gal4 sufficiency were *cut(ue*)-Gal4 > *Myc*; GFP and *cut(ue)*-Gal4 > mRFP; *lov* RNAi. As shown in Fig 3, *cut(ue)*-Gal4 > UAS-*Myc*; *lov* RNAi larvae show behavior that is indistinguishable from that of *cut(ue)*-Gal4 > *lov* RNAi larvae, failing to burrow into food and tunnel into agar plates. This finding indicates that the action of *lov* is epistatic to that of *Myc* in the tracheae.

**Fig 4:**
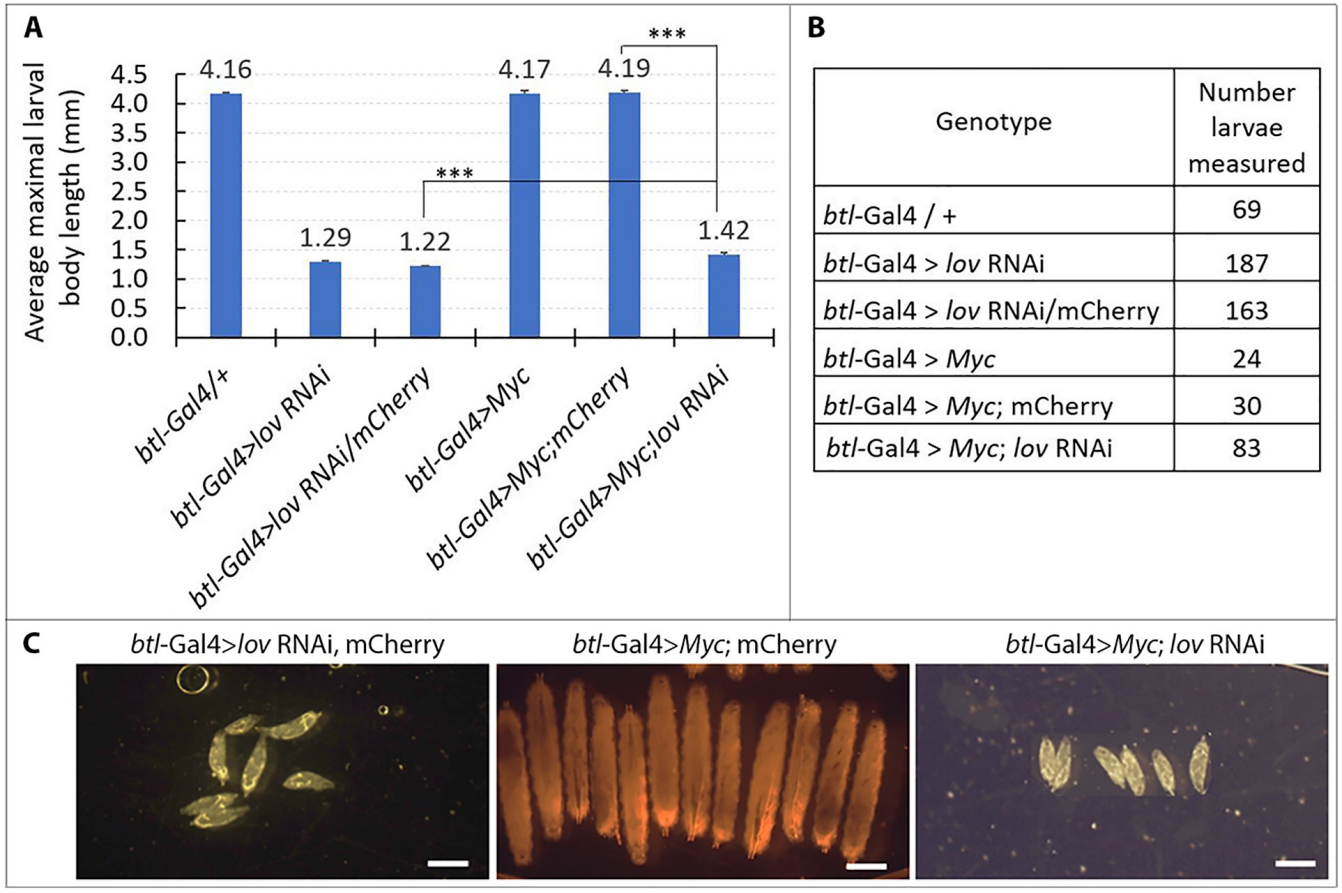
Inhibition of larval growth and survival by *lov* knockdown in the tracheae is epistatic to overexpression of *Myc*. A. Larval lengths at death or pre-pupation. For the measurement protocol see Material and methods. Control *btl* Gal4 /+ larvae and *btl*-Gal4 larvae overexpressing *Myc* or *Myc* with mCherry (control genotype for comparison to *Myc*; *lov* RNAi) survive to pupate and were measured at six days AEL. *btl*-Gal4 > *lov* RNAi, *btl* Gal4 > *lov* RNAi/mCherry and *btl*-Gal4 > *Myc*; *lov* RNAi larvae were measured at death (two-three days AEL). Larvae expressing both *lov* RNAi and *Myc* grew and survived only marginally better than larvae expressing *lov* RNAi alone. ***—p values from Student’s t test for the two critical comparisons are p = 2 x10^-12^ (*lov* RNAi/mCherry versus *Myc*; *lov* RNAi) and 2 x10^-58^ (*Myc*; mCherry versus *Myc*; *lov* RNAi). Error bars = SEMs. **B**. Larval numbers for the analyses. **C**. Representative larvae for the three critical genotypes in A. Direct white light tungsten illumination was used for imaging. This produced variable coloration at different lamp intensities. Malpighian tubules and gut are visible in the larvae in A. and C. Scale bars = 1mm.

The epistasis of the *lov* RNAi effects over those of *Myc* was confirmed by experiments using *btl*-Gal4 to drive the same five constructs in the tracheae and quantifying larval growth and survival through measurements of maximal length at death, or at pupation (Fig 4). When *lov* expression is inhibited throughout the entire tracheal system with *btl*-Gal4, the fluid entry associated with failed EP growth produces hypoxia, which strongly inhibits the overall growth and survival of the larvae. *btl*-Gal4 > *lov* RNAi individuals grow very little and die by three days AEL whereas normal larval growth is associated with a substantial (7–8 fold) increase in length (Fig 4A). Although *btl*-Gal4 > *Myc* larvae have enlarged tracheal cells and nuclei and are noticeably fatter than controls, their overall growth and survival is similar to that of controls (Fig 4A). However, larvae simultaneously expressing *Myc* and *lov* RNAi in the tracheae proved to be thin and died with a length only slightly greater than that of *btl*-Gal4 > *lov* RNAi larvae (Fig 4A). This study thus further demonstrates that *lov* acts downstream to *Myc* in the tracheae with its action effectively blocking that of *Myc*.

**Fig 5:**
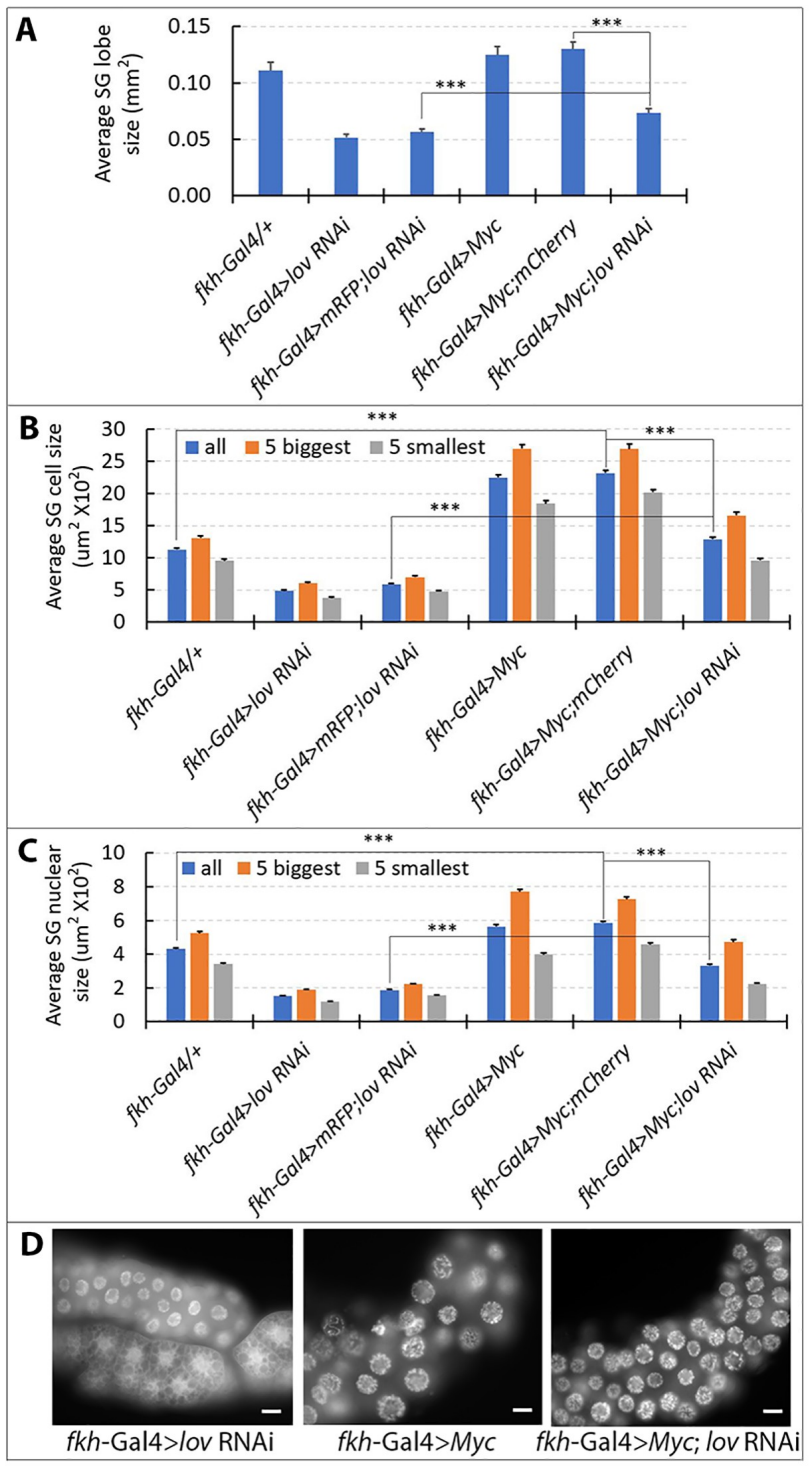
Reducing *lov* expression partially inhibits *Myc* induced EP growth in the salivary glands. *fkh*-Gal4 was used to express *lov* RNAi, *Myc*, or *Myc* and *lov* RNAi together in the salivary glands. As for other Gal4 lines, two control genotypes, mRFP; *lov* RNAi and *Myc*; mCherry, each with 15 UAS sequences total, were examined for comparison to the *fkh*-Gal4 > *Myc*; *lov* RNAi genotype. Details of the measurement protocols are in the Material and Methods. A. Salivary gland lobe size. Individual salivary gland lobes (9–11 per genotype) were measured. Overexpressing *Myc* cannot overcome the reduced salivary gland growth induced by depleting *lov* expression. *** = Student’s t text p values of 3x10^-3^ or lower. B. Salivary gland cell size. The five largest and five smallest cells in the distal one third of individual glands for each genotype were measured. Lobes from at least seven larvae were examined and 98–130 cells were quantitated. Averages for the largest, smallest, and combined total, cells are shown. *lov* depletion partially inhibits the overgrowth produced by *Myc* over-expression.*** Student’s t test p values of 2x10^-40^ or lower. C. Salivary gland nuclear size. Analysis of nuclear size was performed as for the analysis of salivary gland cell size. Between 202–379 nuclei were examined for the each of the various genotypes. The effects of *lov* RNAi on *Myc* overexpression are comparable to those on cell size. *** Student’s t test p values of 2.7 x10^-55^ or lower. Error bars = SEMs. For all three data sets, Student’s t test p values for comparison of *fkh*-Gal4 > + to *fkh*-Gal4 > *lov* RNAi or *fkh*-Gal4 > mRFP; *lov* RNAi were below 8 x10^- 6^. D. Representative nuclear images for the critical comparisons in C. The lower tissue in the *fkh*-Gal4 >*lov* RNAi image is the fat body attached to the salivary gland. Scale bars = 20 μm.

For salivary glands, we examined the effects of the combined *Myc* over-expression and *lov* under-expression phenotypes on three parameters of the tissue—individual lobe size, cell size and nuclear size (Fig 5). Given that the level of ploidy varies approximately two-fold across the gland, with the proximal cells showing a lower level of ploidy [8, 36] we limited our measurements to the distal one third of each gland. Co-expressing *lov* RNAi with *Myc* markedly inhibited the enhanced growth produced by *Myc* alone on all aspects of the tissue (Fig 5A–5C), indicating that *lov* is also required for much of the downstream activity of *Myc* in this organ.

**Fig 6:**
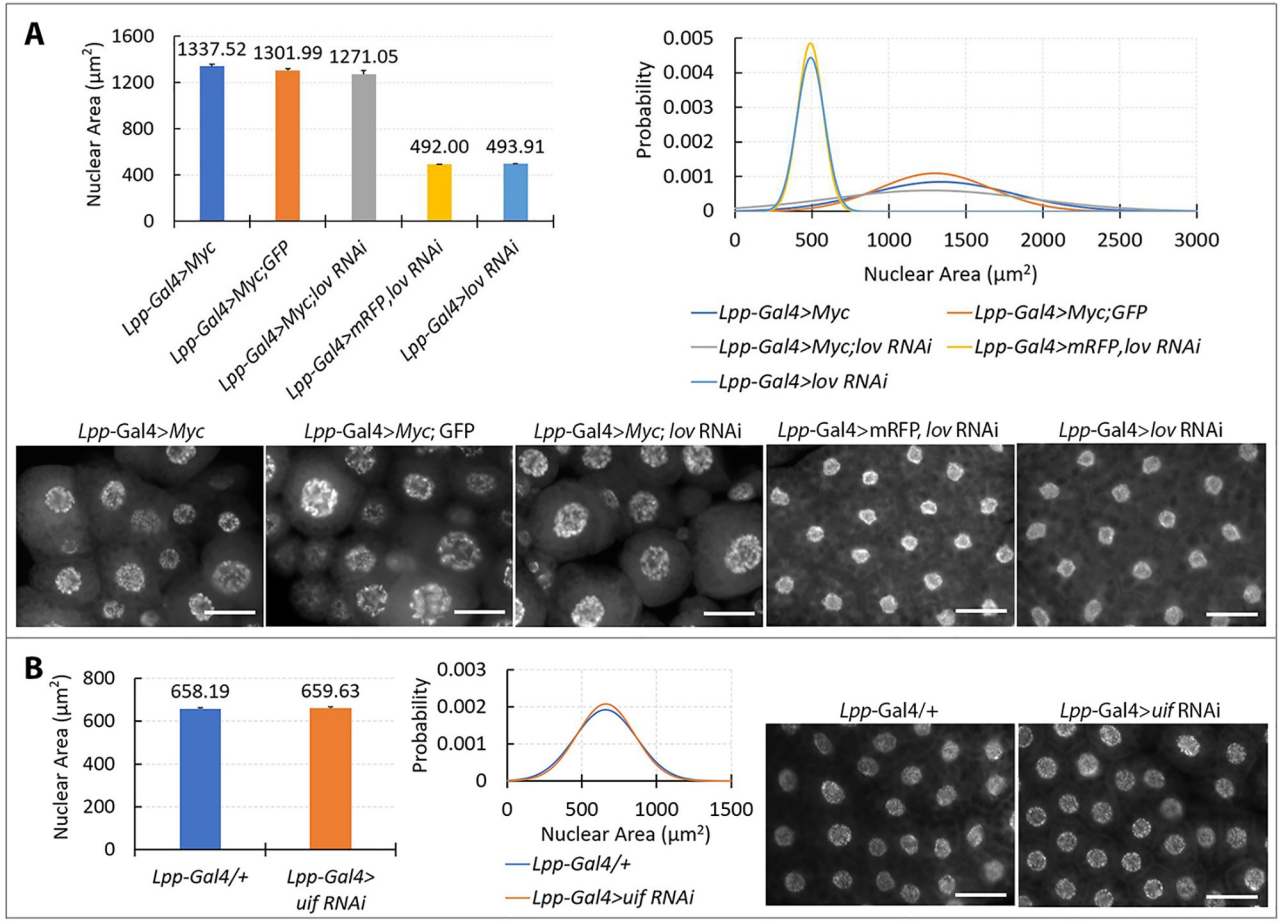
The roles of *lov* and *uif* in fat body EP growth. A. *Myc* over-expression is epistatic to *lov* knockdown in the fat body. The upper left panel shows mean nuclear areas for fat body cells expressing *lov* RNAi, *Myc*, or both constructs, using the *Lpp*-Gal4 fat body driver. Data for two control genotypes (*Lpp*-Gal4 > mRFP; *lov* RNAi and *Lpp*-Gal4 > *Myc*; GFP) that carry the same UAS copy number as the *lov* RNAi; *Myc* test genotype are shown. The means for the *Myc*; GFP and *Myc*; *lov* RNAi genotypes are not statistically different from the *Myc* genotype mean (p = 0.21 and p = 0.07 respectively). The upper right panel shows the distribution of nuclear sizes for the same five genotypes. For the y axis, Probability represents the probability of nuclei of that particular size in the tissue, as graphed in 1 micron2 increments. Note the data for the *lov* RNAi tissue and mRFP; *lov* RNAi tissue are almost perfectly superimposed and therefore hard to distinguish in the graph. For each genotype, fat body from 10 larvae and 500 nuclei total were examined. Lower panels show images of fat body cells with DAPI stained nuclei for all five genotypes. Error bars = SEMs. Scale bar = 50 μm. B. *uif* knockdown has no effect on nuclear size in the fat body. The left panel shows means and standard deviations for nuclear areas in control fat body tissue and tissue expressing *uif* RNAi. The middle panel shows distributions of nuclear sizes as for Fig 8A. The right panels show images of DAPI-stained control and *uif* RNAi fat body tissue. For each genotype, tissue from 10 larvae was examined and 500 nuclei were measured. Scale bar = 50 μm.

In the fat body, given our initial findings (see above) we focused on nuclear changes when examining epistasis between *Myc* and *lov*. *Myc* over-expression alone produced a much broader range of nuclear sizes in this tissue than seen in the tracheae and salivary glands (Fig 6A). In the salivary gland, differences in nuclear area of two-fold were seen on over-expressing *Myc* (Fig 5C), whereas nuclear areas differed over at least an eight-fold range in the fat body (Fig 6A). To compare this phenotype to those of *lov* RNAi and *Myc*; *lov* RNAi and the appropriate controls, we used Imaris software (see Material and methods) to analyze 500 nuclei of each genotype (Fig 6A). This approach quantified the striking increase in the range of nuclear sizes produced by UAS-*Myc* tissue as compared to *lov* RNAi. Surprisingly, it also established that the profile of nuclear sizes for *Myc*; *lov* RNAi tissue is identical to that of the tissue expressing UAS-*Myc* alone (Fig 6A). Thus in this tissue, in contrast to the tracheae and salivary glands, *Myc* is epistatic to *lov*.

In contrast to the tracheae, where *lov* knockdown affects both growth and behavior of the whole organism, *lov* knockdown in the fat body had no obvious effects on larval behavior or growth. *Myc* fat body tissue was detectably more fragile than control, with *Lpp*-Gal4 *Myc*; *lov* RNAi tissue proving even more easily fragmented.

### Tissue specific regulation of *uninflatable* (*uif*) by *lov* contributes to tissue specific differences in EP growth

**Fig 7:**
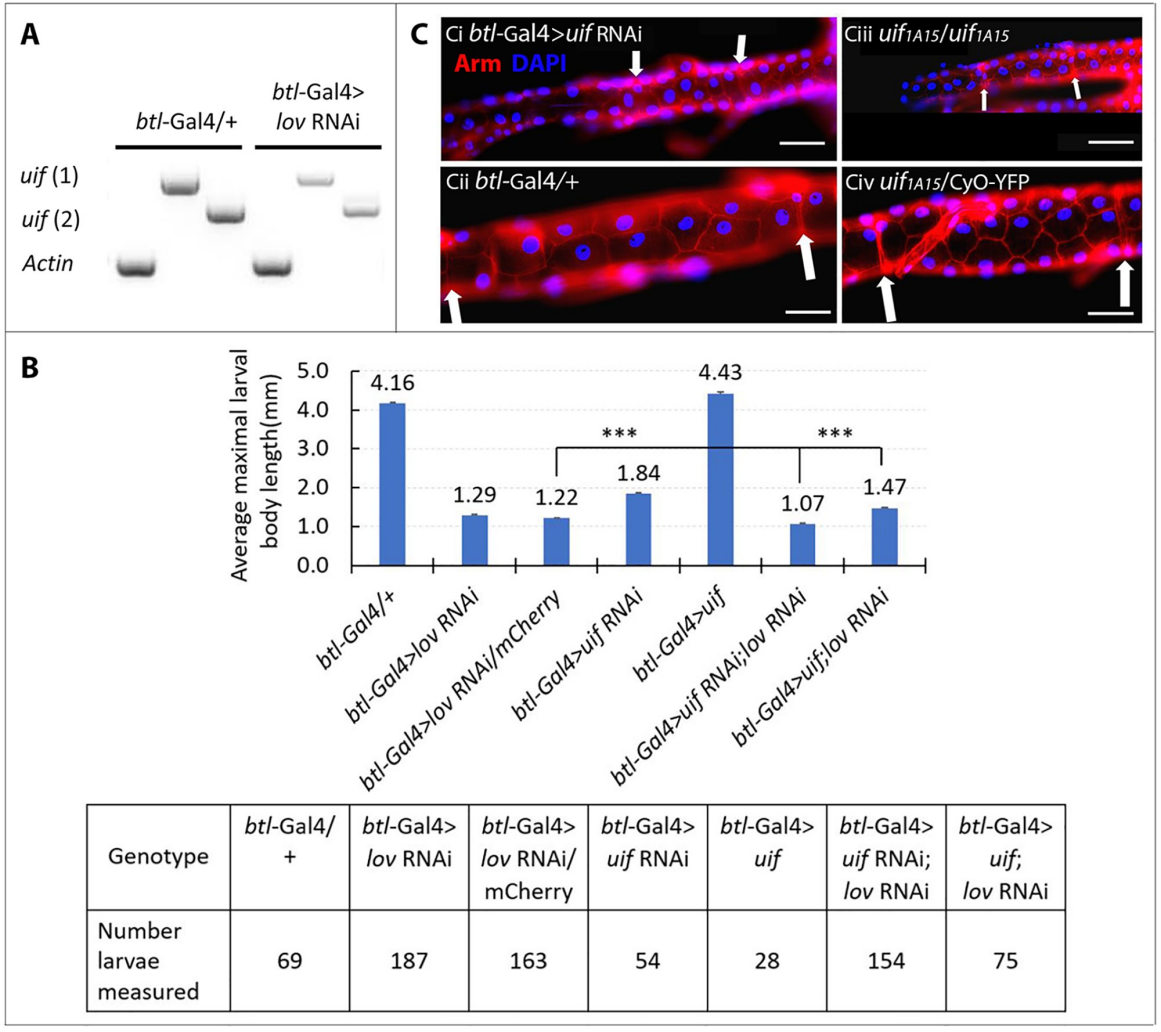
*uif* is positively regulated by *lov* in the tracheae and required for tracheal EP growth. A. Semi Q RT-PCR analysis of *uif* transcript levels in larvae with *lov* knockdown in the tracheae. RNA preps of whole larvae were analyzed. *uif* 1 and *uif* 2 represent *uif* transcript products detected with two different sets of *uif* primers. Actin 57B primers were used for the control. *btl*-Gal4 only expresses *lov* RNAi in the tracheae, but decreased *uif* RNA levels are clearly detectable in these whole body samples. B. Using larval length measurement as a readout for tracheal growth indicates that *lov* acts upstream of *uif* in the tracheae. Two control genotypes (*btl*-Gal4/+, *btl*-Gal4 > *uif*) survive the larval stages to pupate and their lengths were measured at six days after egg lay (AEL). *btl*-Gal4 > *lov* RNAi larvae die by three days AEL and all genotypes to be compared to these larvae were measured at three days AEL. The growth inhibition for *btl*-Gal4 > *lov* RNAi; mCherry larvae (20 copies of UAS total) is slightly greater than that for *btl*-Gal4 > *lov* RNAi larvae (10 copies of UAS total) demonstrating that Gal4 levels from the *btl* construct are not limiting. Student t tests showed highly significant differences for all possible pairs of genotypes with p values ranging from 9x10^-9^ to 8x10^-45^. For the two critical comparisons to *btl*-Gal4 > *lov* RNAi, labelled ***, p = 1x10^-14^ (*btl*-Gal4 > *uif* RNAi; *lov* RNAi) and 9x10^-9^ (btl-Gal4 > *uif*; *lov* RNAi) Error bars = SEMs. C. Loss of *uif* expression in the tracheae inhibits EP growth. Tracheal metamere 9, defined by its flanking fusion cells (white arrows) is shown for the following genotypes: *btl*-Gal4 > *uif* RNAi (Ci), *btl*-Gal4/+ (Cii), homozygous *uif*^*1A15*^ (Ciii) and *uif*^*1A15*^ / *CyO*-YFP all at six days AEL. Cell and nuclear size are both strongly reduced by loss of *uif* expression. Scale bar = 50μm.

The tracheae and fat body perform very different physiological functions and presumably show many differences in gene expression. Although we have shown that one site of action of *lov* is the nucleolus, it seemed possible that *lov* could also act as a transcription factor regulating tissue-specific gene expression in EP tissues. In the tracheae, *lov* knockdown results in extreme shortening of individual metameres, fluid accumulation in the lumen, and tracheal breakage. We therefore investigated genes that regulate processes related to these phenomena. These were *uninflatable* [23] and *pickpocket 4* and *11* [37], which act to remove the fluid from the embryonic tracheae at hatching; *coracle* [38] and *Fas III* [39], which are required for formation of the watertight apical septate junctions and *serpentine*, *vermiform* [40], *krozkopf verkehrt* [41] and *mummy* [42], which are all involved in synthesis of cuticular components required for tracheal growth. We have previously published our finding that Coracle and Fas III proteins are not affected by *lov* knockdown [24]. For six of the other seven genes investigated, tracheal *lov* RNAi knockdown had no reproducible effect on transcript levels, as measured in whole larvae with *lov* knockdown in the tracheae (*btl*-Gal4 > *lov* RNAi larvae). However, *uif* transcript levels were strongly reduced in these larvae (Fig 7A). This finding was confirmed by use of two different sets of *uif* primers for the experiments (Fig 7A). Mutant and RNAi analysis for *uif* has previously shown that this gene functions in overall longitudinal growth of the larval tracheae [23, 26]. Our finding further indicates that *uif* is a downstream target of *lov* in the tracheae and is part of the mechanism by which it regulates EP growth in this tissue.

To investigate this proposed regulation of *uif* by *lov* further, we examined the effects of over- or under-expressing *uif* on *lov* tracheal knockdown, using the *btl*-Gal4 driver and the larval length assay described above (Fig 7B). The *uif* RNAi construct described by Zhang and Ward [23] was used for these experiments. Given the massive size of the Uif protein (~380 kDa), no UAS-*uif* cDNA construct has been generated for overexpression studies. However, a chromosome carrying a UAS insertion into the 5’flank of the *uif* gene has been shown to give weak Gal-4 induced expression of *uif* [43]. This chromosome was used to test for *uif* rescue of *lov* knockdown.

The *uif* RNAi construct produced a weaker effect on overall larval growth than the *lov* RNAi construct when expressed alone. This could reflect a difference in the efficacy of the two RNAi constructs or indicate a more extensive role for *lov* in tracheal growth. When the *uif* RNAi was co-expressed with *lov* RNAi, growth/survival inhibition proved additive (Fig 7B), indicating either that *lov* and *uif* act in the same pathway or have independent roles in the trachea. The genomic UAS *uif* over-expression construct had a small effect on larval length when expressed alone in the tracheae and gave a highly statistically significant partial rescue of the effects of *lov* RNAi, increasing larval length by 20% (Fig 7B). Complete rescue of the *lov* knockdown would be unexpected given i) the weak *uif* expression associated with the construct and ii) the likelihood that *lov* regulates other tracheal genes. These findings thus argue strongly that *lov* positively regulates *uif* expression, acting upstream of *uif* in the same pathway for tracheal growth.

We confirmed the further prediction that *uif’s* role in trachea is specifically to promote EP growth by cytological examination of the tracheae in *btl*-Gal4 > *uif* RNAi larvae and larvae homozygous for the weak *uif* mutation, *uif*^*1A15*^ [23]. Because both the *btl*-Gal4 > *uif* RNAi and the *uif*^*1A15*^ genotypes produce weaker effects on the tracheae than *btl*-Gal4 > *lov* RNAi, we could examine their effects throughout the entire dorsal trunk tracheae as opposed to the limited region of *cut* (*ue*)-Gal4 expression. Fig 7C shows metamere 9 of the dorsal trunks from control, *btl*-Gal4 > *uif* RNAi, and homozygous *uif*^*1A15*^ larvae. *uif* knockdown by either route dramatically reduces the size of tracheal epithelial cells and their nuclei. The similarity of the *btl*-Gal4 > *uif* RNAi phenotype to the homozygous *uif*^*1A15*^ phenotype confirms the specificity of the *uif* RNAi construct.

**Fig 8:**
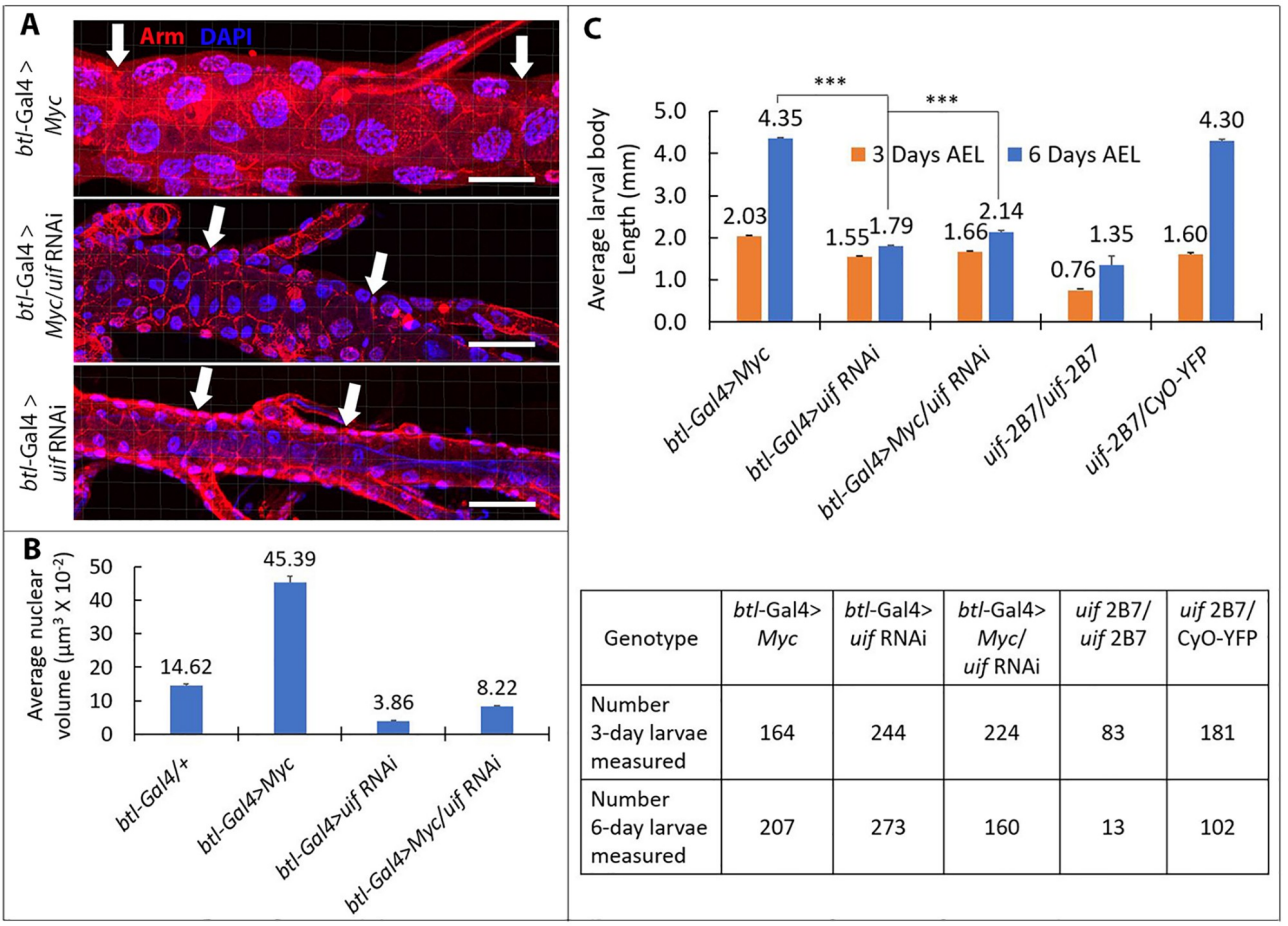
uif acts downstream of Myc in tracheal EP growth. A. Confocal images of tracheal metamere 9 for larvae expressing Myc, uif RNAi, or both constructs with the btl-Gal4 driver. White arrows indicate the limits of metamere 9 as defined by the flanking fusion cells. Scale bar = 50 μm. B. Nuclear volumes for the genotypes in A and a further control (btl-Gal4 > +, see Fig 1). Volumes were calculated as described in Material and Methods using Imaris software. Six- eight individual tracheae and 101–222 nuclei were examined for the various genotypes. Error bars = SEMs. C. Larval lengths were measured at three and six days for the genotypes examined in A and for larvae homozygous and heterozygous for the uif 2B7 null mutation. More than 80% of the uif2B7 homozygous larvae died between three and six days and therefore only a small number were measured at six days. *** Student’s t p values of 2.76x10-272 (btl-Gal4 > Myc versus btl-Gal4 > uif RNAi and 4.09x10-180 (btl-Gal4 > Myc; uif RNAi).

As a target of *lov* action in the tracheae, *uif* knockdown would also be predicted to act genetically downstream of *Myc*, like *lov* itself. We investigated this possibility using the opposing tracheal phenotypes of *uif* RNAi and UAS-*Myc*, as described above. Larval length measurements revealed that *btl*-Gal4 > *Myc*/*uif* RNAi larvae show almost as much inhibition of growth as *btl*-Gal4 > *uif* RNAi larvae (Fig 8C) indicating a downstream action of *uif* relative to *Myc*. We included larvae carrying a null mutation of *uif*, *uif*^*2B7*^ [23], in these length measurements to provide a further comparison to the effects of the *uif* RNAi construct. As shown, loss of *uif* function throughout the larvae is significantly more deleterious than *uif* knockdown in the tracheae alone.

Examination of cell and nuclear size in metamere 9 for tracheae expressing *Myc*, or *uif* RNAi, or both constructs (Fig 8A and 8B) confirmed that loss of *uif* expression produces dramatic inhibition of the EP growth-promoting effects of *Myc* over-expression. Together these various results indicate that in the trachea, regulation of *uif* expression is a critical function of *lov*, acting downstream of *Myc* in the regulation of the EP growth of the tracheae.

Uif protein is primarily found on the apical surface of the tracheal epithelial cells [23], which is the site of continuing cuticular growth during the larval phase. Uif is also strongly expressed in other ectodermally derived organs particularly those with a prominent cuticle like the epidermis and adult wings [44]. In contrast the fat body is a mesodermal tissue and these are not indicated to express *uif*. It seemed likely therefore that a difference in *uif* expression might contribute to the differing epistatic relationships between *Myc* and *lov* in the tracheae and fat body. To test this possibility, we determined whether *uif* knockdown could suppress EP growth in the fat body. As shown in Fig 6B *uif* knockdown has no effect on fat body EP growth. We conclude that the difference in expression of *uif* between the tracheae and the fat body is a component of the differing role(s) of *lov* in the two tissues.

## Discussion

### Different EP growth regulation mechanisms operate in different larval tissues

Our studies indicate a role for *lov* in the growth of all the EP tissues investigated. *Myc*, like *lov*, also has a universal role in EP growth but the difference in *uif* function between the tracheae and the fat body leads to a difference in the relationship of *lov* and *Myc* in the two tissues. *lov* is epistatic to *Myc* in the tracheae because the action of one of *lov*’s tracheal targets, *uif*, is downstream of *Myc*. But in the fat body, in the absence of a role for *uif*, loss of *lov* function has much smaller effect on *Myc*-induced enhanced EP growth.

Three of the four EP tissues we have investigated (tracheae, salivary glands, epidermis) are epithelial tissues of ectodermal origin. In the two of these tissues (tracheae and salivary glands), *lov* acts downstream of *Myc*. Given that the downstream action of *lov* in one of these tissues involves its target, *uif*, we considered the possibly that the EP growth of all ectodermally-derived larval tissues is dependent on *uif* expression regulated by *lov*. The epidermis is known to express *uif* in larval life, but *uif* expression in the salivary gland is limited to the embryonic stage [23]. Other *lov* targets are therefore likely to act downstream of *Myc* in the larval salivary gland.

The fat body is derived from the mesoderm, and the cellular effects of *lov* knockdown in this tissue clearly differ from those in the three ectodermally derived tissues studied. *lov* plays a lesser role in EP growth with a smaller effect on nuclear size and no detectable effect on cell size (Fig 1D and 1G). *uif* knockdown has no effect on fat body EP growth and in contrast to the other tissues, *lov* acts upstream of *Myc* in the fat body. It is possible that the action of *lov* in this tissue involves regulation of tissue-specific genes with very minor roles in EP growth. An alternative explanation involves *lov’s* action at the nucleolus. We have detected Lov protein in the nucleoli of multiple EP tissues suggesting that its role at this site is shared between many tissues. The action of *lov* relative to *Myc* in the fat body could thus represent the phenotype of *lov* knockdown when only its nucleolar function is operating in a tissue.

Given that Lov and Myc are both transcription factors one possible underlying mechanism for their interactions would be transcriptional regulation of one gene by the other. We have examined the effects of under- or over-expressing each of them on the transcript levels of the other using whole larval RNA preparations and have found no evidence of cross-regulation at this level (unpublished findings). But these data represent a summation of events in all the individual tissues, which could easily obscure tissue-specific responses. A tissue-by-tissue approach will be necessary to fully address this question.

Altogether our studies of the relationship between *lov* and *Myc* demonstrate heterogeneity in the responses of individual EP tissues to *lov* as a regulator of EP growth. As such, they emphasize that EP growth cannot be viewed as a single universal mechanism under the control of the master regulator *Myc*, but rather as a variable phenomenon, in which tissue-specific growth factors modulate *Myc* action.

### The molecular function of Lov

Some insights into how Lov might act at the molecular level can be gained by considering the functions of its close structural relatives. Lov is a member of the Tramtrack (Ttk) BTB/POZ protein subfamily in *Drosophila*, almost all of whose members also contain at least one DNA binding motif. These proteins are distinctive in that they show unusually high sequence similarity in three regions of the BTB/POZ domain that form an interface for protein-protein binding [21]. This similarity led to the prediction that Ttk proteins generate a network of interacting DNA binding proteins, whose functions require partnering through their BTB/POZ domains. Multiple studies have confirmed this hypothesis. Complexes of Ttk proteins have been shown to regulate both chromatin-related functions [45–47] and quantitative aspects of certain developmental processes [48, 49].

These findings thus argue for role(s) for Lov in chromatin structure that involve interaction with other Ttk protein group members. Interestingly Tramtrack has been shown to have a role in the signaling pathway that initiates EP growth in the ovarian follicle cells [50], where it acts downstream of the cell surface receptor Notch. Tramtrack thus represents one potential binding partner for Lov. No other Ttk family member has been shown to localize primarily to the nucleolus like Lov but Ribbon is known to be required for nucleolar integrity in the larval salivary glands (Rajprasad Loganathan, personal communication) suggesting that it too could be a Lov interaction partner.

Formation of homotypic and heterotypic dimers and oligomers of Lov with other Ttk proteins may also underlie our unexpected discovery that over-expressing *lov* in various EP tissues produces a more severe version of the *lov* knockdown phenotype (see Results). Over-expressing *lov* could disrupt its interaction patterns with other Ttk proteins causing loss of its normal activities. Studies to address these possibilities are in progress.

### The role of *uif* in larval EP growth

The signals in late embryogenesis that initiate the transition to EP growth in preparation for larval life have not been identified, but for initiation of EP growth in the somatic follicle cells of the adult ovary, the activating sequence has been well-characterized. Upregulation of the Notch ligand Delta in the adjacent germ line cells leads to Notch activation in the follicle cells followed by Notch generated transcriptional events that block the mitotic G2-M transition and promote the G1-S transition of the cell cycle [51, 52]. *Myc* over-expression is not capable of initiating a switch from mitotic to endocycling in these cells but rather it accelerates the endocycle so that larger cells with larger nuclei are produced [52]. These findings for the role of *Myc* are consonant with our current knowledge of *Myc*’s role in EP growth in larval cells: it appears to be a downstream activator that coordinates enhanced cellular growth.

In the trachea, our findings indicate that *uif* acts late in the chain of events that promote EP growth, as a downstream target of the transcription factor Lov, which is itself downstream of the transcription factor Myc. This late function suggests an intracellular role for Uif, but both its structure and cellular location seem at odds with this possibility: Uif is a transmembrane protein of the apical plasma membrane of the tracheal epithelial cells. Its extracellular domain contains a continuous array of 14 EGF-like repeats. This structure evokes the stretches of EGF-like repeats in the extracellular domains of Notch and its ligands Delta and Serrate, suggesting a role for Uif as a membrane signaling molecule that interacts with Notch or another EGF-like repeat containing protein at the cell surface.

Two studies have addressed the significance of the EGF-like repeats of Uif in relation to Notch function [43, 53]. Both found that Uif has no role in the classic plasma membrane signaling function of Notch and currently there is no evidence that the extracellular domain of Uif acts as a receptor to transduce an extracellular signal. However, Loubery et al. [53] have identified a novel intracellular interaction of Uif with Notch required for the asymmetric distribution of Notch on Sara endosomes during sister socket/sheath cell differentiation. Uif and Notch are internalized independently to these endosomes but their interaction on the endosome surface, through four EGF-like repeats in the Uif external domain, determines the asymmetric inheritance of the Sara endosomes. Intracellular endosome trafficking of Uif has also been demonstrated in the tracheae [54, 55], with Uif co-localizing internally with Crumbs, another apical transmembrane protein that contains EGF-like repeats.

These intracellular actions of Uif and the known role of Notch in initiating follicle cell EP growth suggest a possible regulatory pathway for activation of tracheal EP growth. A receptor with EGF-like repeats could be the trans-membrane activator, with Uif’s role involving internalization onto a population of endosomes prior to interaction with this protein. Such a site of action for Uif is more consonant with our discovery that *uif* acts downstream of *lov* and *Myc* in the tracheae than a role for Uif in plasma membrane initiation of EP growth.

## Supporting information

S1 Table Sources for fly stocks used. (PDF)

S1 Fig *lov* null mutant larvae show similar tracheal damage to *btl*-Gal4 > *lov* RNAi larvae. We have identified three transposon insertions into the *lov* gene that are protein null mutations (manuscript in preparation). They all produce the same larval phenotype: embryogenesis appears normal but larvae die shortly before or after hatching. Larvae that survive hatching show tracheal phenotypes that are similar to those produced by expressing *lov* RNAi throughout the tracheal system with *btl*-Gal-4 (Zhou et al. PLOS ONE 2016; 11(8): e0160233) but the tracheal damage appears earlier in *lov* null mutants and is associated with earlier death. Images of larvae heterozygous and hemizygous for one of these mutations (*lov*^*M102458*^—see Flybase page for *jim lovell* gene) are shown. **A. Control (*lov***^***M102458***^**/CyO-GFP) larva**. Anterior region of a one day old larva. The dorsal trunk tracheae (**dts**) and other tracheal branches have expanded and are air-filled, which makes them highly visible in the larval body. **B. Hemizygous (*lov***^***M102458***^**/*Def(2R) K10* larva**. *Def(2R) K10* removes *lov* and several other genes (Duman-Scheel et al. Development 124, 2855–2865, 1997). Anterior region of a one day old larva. The dorsal trunks are noticeably narrower than those of control larvae, and long stretches of the trunks (see the red lines) are almost undetectable because they are filled with fluid. **cpa** = cephalopharyngeal apparatus. Scale bars = 50 m.
(DOCX)

S1 Raw image (DOCX)
