# Supplementary material for: The roles of jim lovell and uninflatable in different endopolyploid larval tissues of Drosophila melanogaster
Source: PLoS One. 2020 Aug 21;15(8):e0237662. doi: 10.1371/journal.pone.0237662 (PMC7444548; doi:10.1371/journal.pone.0237662)
Supplement: S1 Table — (PDF) [file pone.0237662.s001.pdf]

Construct Stock	Source	Comment
cut(ue)-Gal4	BDSC BL27327	
btl-Gal4 on II	Lan Jiang	Ghabral et al. PLOS Genetics 7, e1002087, 2011
btl-Gal4 on III	Lan Jiang	Same construct/origin as btl-Gal4 on II
da-Gal4	BDSC BL55850	
hs-Gal4	BDSC BL1799	
fkh-Gal4	BDSC BL78060	
ppl-Gal4	BDSC BL58768	
Lpp-Gal4	Suzanne Eaton	Palm et al. PLOS Genetics 8, e1002828., 2012
A58-Gal4<br>UAS-src-GFP, UAS-DsRed2-Nuc, A58-Gal4/TM6B	Michael Galko	Lesch C. et al. Genetics 186, 943-957,2010
stripe-Gal4	Michael Galko	Subramanian et al. Current Biology 13, 1086-1095, 2003
10x UAS-lov RNAi	TRiP HMSO1126	
10x UAS-uif RNAi	VDRC v1050	
10x UAS-Myc RNAi	BDSC BL36123	
5xUAS-lov	Beckingham Lab	Bjorum et al. PLOS ONE 8: e61270.
5xUAS-uif	DGRC-Kyoto Stock Ctr 203-493	
5xUAS-Myc	BDSC BL9674	
5xUAS-mir-mRFP	BDSC BL7118	
10xUAS-GFP pJFRC28	Herman Dierick	Pfeiffer et al. PNAS 109, 6626-6631, 2012
10xUAS-GFP 10xUAS-IVS-mCD8-GFP	BDSC BL32185	
Bac-Myc-GFP	BDSC BL38633	
TubP-Gal 80, Tb/ TM3 Sb	BDSC BL36283	
C(1)DX/YBs; RFP-Fib; EGFP-Mod	Eric Wieschaus	Falahati & Wieschaus PNAS 114, 1335-1340, 2017
C(1)DX/YBs; RFP-Fib; EGFP-NS1	ibid	ibid
C(1)DX/YBs; RFP-Fib: Pit-EGFP	ibid	ibid
C(1)RM/0; Rpl 135-EGFP	bid	ibid
w; uif^{2B7}/CyO YFP	Robert E. Ward III	Zhang & Ward Dev Biol 336 201-212 2009
w; uif^{1A15}/CyO YFP	ibid	ibid
