# Supplementary figures and images for: The roles of jim lovell and uninflatable in different endopolyploid larval tissues of Drosophila melanogaster
Source: PLoS One. 2020 Aug 21;15(8):e0237662. doi: 10.1371/journal.pone.0237662 (PMC7444548; doi:10.1371/journal.pone.0237662)

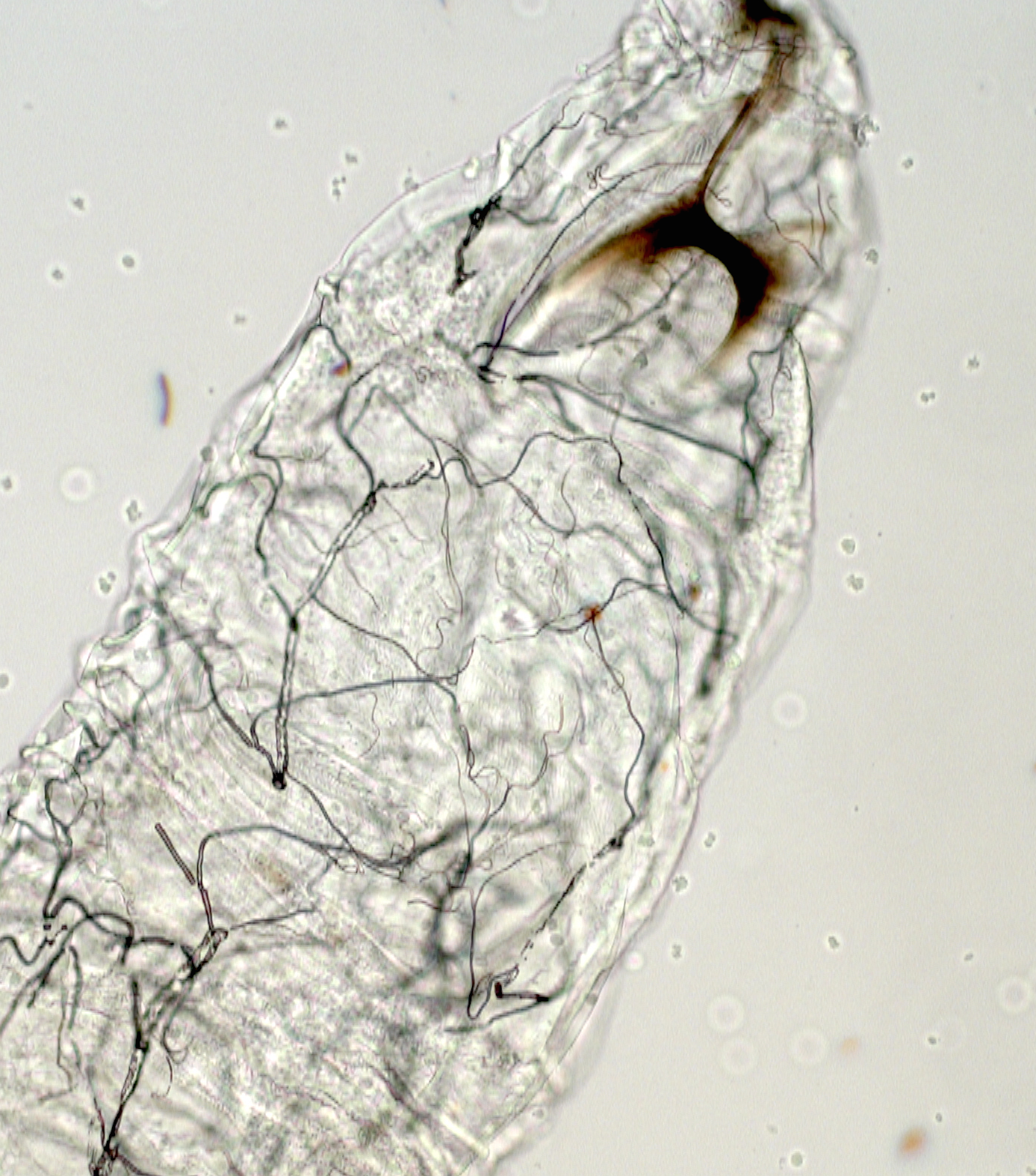

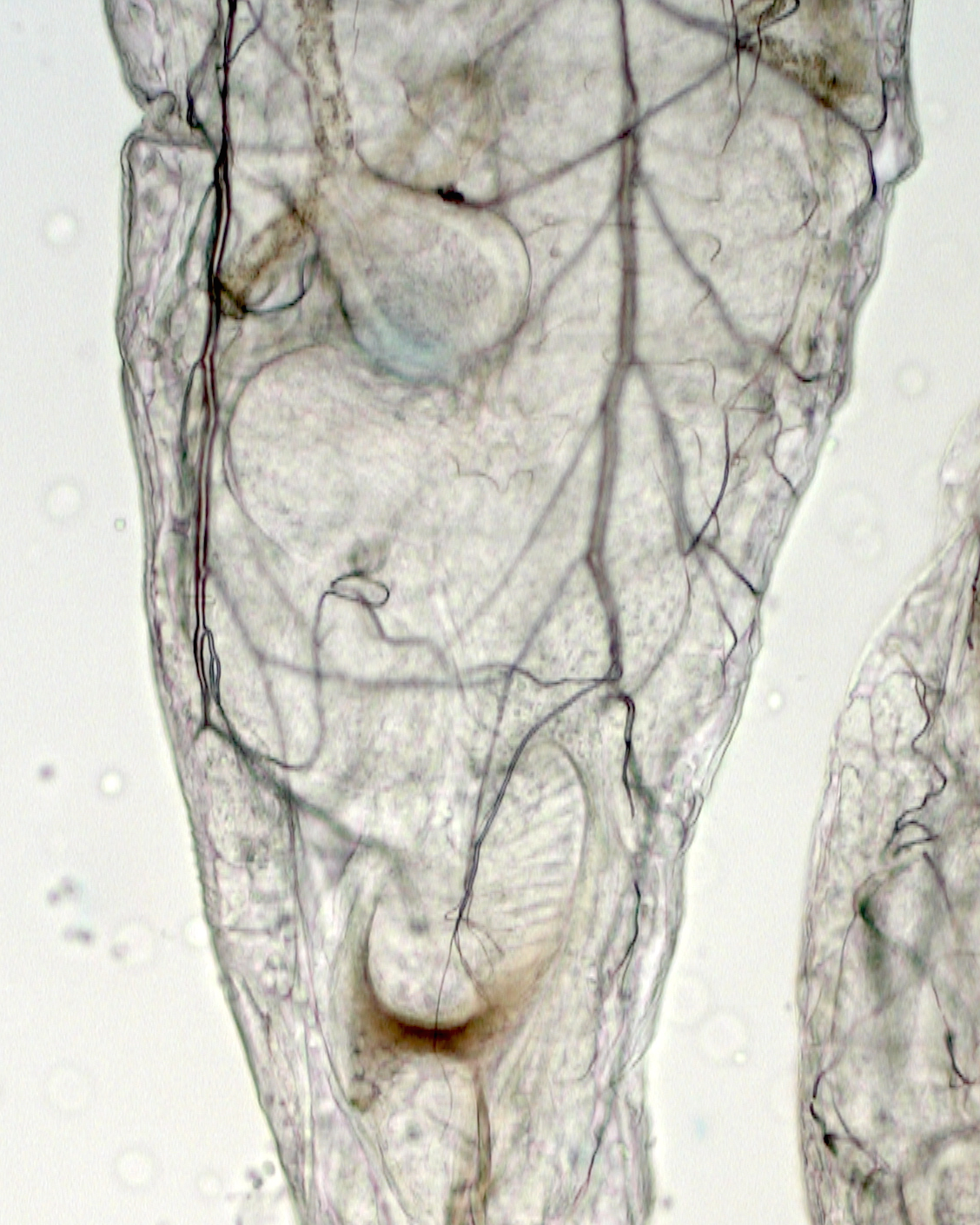


dts

dts

cpa

cpa

**A**

**B**

Zhou et al. PONE-D-20-09345 Supplemental Figure 1

Supplement: S1 Fig — We have identified three transposon insertions into the lov gene that are protein null mutations (manuscript in preparation). They all produce the same larval phenotype: embryogenesis appears normal but larvae die shortly before or after hatching. Larvae that survive hatching show tracheal phenotypes that are similar to those produced by expressing lov RNAi throughout the tracheal system with btl-Gal-4 (Zhou et al. PLOS ONE 2016; 11(8): e0160233) but the tracheal damage appears earlier in lov null mutants and is associated with earlier death. Images of larvae heterozygous and hemizygous for one of these mutations (lovM102458—see Flybase page for jim lovell gene) are shown. A. Control (lovM102458/CyO-GFP) larva. Anterior region of a one day old larva. The dorsal trunk tracheae (dts) and other tracheal branches have expanded and are air-filled, which makes them highly visible in the larval body. B. Hemizygous (lovM102458/Def(2R) K10 larva. Def(2R) K10 removes lov and several other genes (Duman-Scheel et al. Development 124, 2855–2865, 1997). Anterior region of a one day old larva. The dorsal trunks are noticeably narrower than those of control larvae, and long stretches of the trunks (see the red lines) are almost undetectable because they are filled with fluid. cpa = cephalopharyngeal apparatus. Scale bars = 50 m. (DOCX) [file pone.0237662.s002.docx]
