# Supplementary material for: The roles of jim lovell and uninflatable in different endopolyploid larval tissues of Drosophila melanogaster
Source: PLoS One. 2020 Aug 21;15(8):e0237662. doi: 10.1371/journal.pone.0237662 (PMC7444548; doi:10.1371/journal.pone.0237662)
Supplement: S1 Raw image — (DOCX) [file pone.0237662.s003.docx]

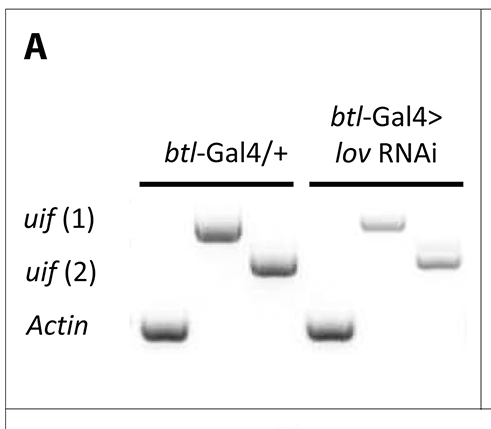


**ORIGINAL GEL IMAGE FOR FIGURE 7A**

PONE-D-20-09345
The roles of Jim Lovell and Uninflatable in different endopolyploid larval tissues of *Drosophila melanogaster* PLOS ONE

This is the only gel image in the manuscript. The gel is an agarose gel of PCR fragments generated by Semi Q RT-PCR from Drosophila larval RNA samples of the genotypes indicated. *uif* (1) and *uif* (2) represent the PCR products produced by two different pairs of primers for the *uif* coding sequence. The gel was stained with Gel Red (Phenix Research) and imaged with a Biorad Universal Hood II gel documentation system. The samples shown are the only samples that were run on the gel. The software of the gel documentation system was used reverse black and white in the image and the image was trimmed to remove unused parts of the gel.
